# Crystal structure of PMGL2 esterase from the hormone-sensitive lipase family with GCSAG motif around the catalytic serine

**DOI:** 10.1371/journal.pone.0226838

**Published:** 2020-01-28

**Authors:** Konstantin M. Boyko, Marya V. Kryukova, Lada E. Petrovskaya, Alena Y. Nikolaeva, Dmitry A. Korzhenevsky, Ksenia A. Novototskaya-Vlasova, Elizaveta M. Rivkina, Dmitry A. Dolgikh, Mikhail P. Kirpichnikov, Vladimir O. Popov

**Affiliations:** 1 Department of Enzyme Engineering, Bach Institute of Biochemistry, Research Center of Biotechnology of the Russian Academy of Sciences, Moscow, Russia; 2 Kurchatov Complex of NBICS-technologies, National Research Centre "Kurchatov Institute", Moscow, Russia; 3 Department of Bioengineering, Shemyakin & Ovchinnikov Institute of Bioorganic Chemistry, Russian Academy of Sciences, Moscow, Russia; 4 Laboratory of Soil Cryology, Institute of Physicochemical and Biological Problems in Soil Science, Russian Academy of Sciences, Pushchino, Moscow Region, Russia; 5 Department of Biology, M.V. Lomonosov Moscow State University, Moscow, Russia; Griffith University, AUSTRALIA

## Abstract

Lipases comprise a large class of hydrolytic enzymes which catalyze the cleavage of the ester bonds in triacylglycerols and find numerous biotechnological applications. Previously, we have cloned the gene coding for a novel esterase PMGL2 from a Siberian permafrost metagenomic DNA library. We have determined the 3D structure of PMGL2 which belongs to the hormone-sensitive lipase (HSL) family and contains a new variant of the active site motif, GCSAG. Similar to many other HSLs, PMGL2 forms dimers in solution and in the crystal. Our results demonstrated that PMGL2 and structurally characterized members of the GTSAG motif subfamily possess a common dimerization interface that significantly differs from that of members of the GDSAG subfamily of known structure. Moreover, PMGL2 had a unique organization of the active site cavity with significantly different topology compared to the other lipolytic enzymes from the HSL family with known structure including the distinct orientation of the active site entrances within the dimer and about four times larger size of the active site cavity. To study the role of the cysteine residue in GCSAG motif of PMGL2, the catalytic properties and structure of its double C173T/C202S mutant were examined and found to be very similar to the wild type protein. The presence of the bound PEG molecule in the active site of the mutant form allowed for precise mapping of the amino acid residues forming the substrate cavity.

## Introduction

Microorganisms inhabiting extreme environments (so called extremophiles) are of special interest for biotechnological application because they possess enzymes able to facilitate reactions under some special and often extreme conditions: at elevated or decreased temperature, in the presence of high salinity or organic solvents and so on [1–4]. Functional and structural characterization of such enzymes provides valuable information about the mechanisms of protein conformational stability and enzymatic catalysis. Unfortunately, isolation of extremophilic microorganisms is difficult in many cases and usually requires elaboration of sophisticated methods and cultivation conditions. The rise of the metagenomics era provided a great opportunity to operate directly with DNA isolated from various environmental sources irrespectively of the ability to obtain a pure culture of a given microorganism [5]. Numerous enzymes were cloned and produced as a result of screening of metagenomic DNA libraries, including proteases, cellulases, lipases/esterases and many others [6–8].

Lipases are hydrolytic enzymes which catalyze cleavage of the ester bonds in triacylglycerols [9, 10]. They can be used as biocatalysts in a variety of applications including food industry, detergents manufacturing, biofuel production and fine chemical synthesis [9–11]. All lipolytic enzymes possess a common α/β hydrolase fold and are classified into families I-VIII according to the presence of the conserved sequence motifs [12–14]. Three-dimensional structures of many lipolytic enzymes have been resolved to date [15]. The catalytic domain of the lipases represents a β-sheet of 5–11 β-strands surrounded by α-helices and contains a canonical catalytic triad which is composed of Ser-His-Asp/Glu residues. Ligand access to the substrate binding cavity is regulated by a CAP (or lid) domain which commonly possesses an α-helical fold [12].

Lipolytic enzymes belonging to the Family IV, or hormone-sensitive lipase (HSL) family are frequently isolated from different sources. These enzymes usually prefer short-chain substrates [16]. Several esterases from this family were previously cloned and characterized including enzymes from metagenomic DNA libraries [17]. Common structural features of these esterases include the conserved HGG motif [13, 18], which participates in the oxyanion hole formation, motifs GxSxG, GGRD and FxxGxxHxxF [13, 18–20], containing active site residues as well as YRMP motif [20, 21]. According to the structure of the motif surrounding the catalytic serine residue, members of the HSL family are further divided into two subfamilies with GD**S**AG and GT**S**AG variants of this sequence [18, 19]; the latter subfamily includes different hydrophilic residues, except for Asp/Glu, in the position before the catalytic serine. Most HSLs form dimers or higher oligomers in solution and in crystal [19]. Oligomerization is believed to be important for thermostabilization of these enzymes [22, 23]. In contrast to the enzymes belonging to GDSAG group, the enzymes from GTSAG group have been poorly characterized [18, 19].

Siberian permafrost is a low temperature environment inhabited by diverse microbial communities, which are adapted to the extreme conditions including permanently frozen ground, limited accessibility of organic matter, low water activity and so on [24–27]. Previously, we have produced and studied several lipolytic enzymes from the psychrophilic bacterium *Psychrobacter cryohalolentis* K5T, which was isolated from a cryopeg buried in the permafrost soil [28]. We have revealed different substrate specificities and various temperature optima of these enzymes demonstrating the broad spectrum of enzymatic activities which could be provided by the genome even of a single bacterium [29–32]. Screening of the metagenomic DNA library obtained from the permafrost-derived microcosm lead to the cloning of several genes coding for potential lipolytic enzymes [21, 33]. Among them, a new esterase PMGL2 belonging to the hormone sensitive lipase (HSL, EC 3.1.1.79) family (family IV lipases) was produced and biochemically characterized [21]. The unique feature of this enzyme is the presence of a cysteine residue in the GCSAG motif which surrounds the conserved catalytic serine. In accordance with the canonical division of this enzyme family on two subfamilies with GDSAG and GTSAG variants of the motif and taking into account the fact that there are possible substitutions in amino acid preceding the catalytic serine of GTSAG variants, we decided to assign PMGL2 into GTSAG subfamily. In order to reveal the structural features associated with this new sequence motif, we have crystallized the wild type PMGL2 (wtPMGL2) and determined its 3D structure. It was the third structure of an enzyme from the GTSAG motif subfamily established and the first one with the GCSAG variant. To shed light on a possible role of the cysteine from this motif, we elucidated the structure of a PMGL2 mutant form (mPMGL2), where the two cysteine residues C173 (from the motif) and the adjacent C202 from the active site cavity were substituted by threonine and serine, correspondingly. mPMGL2 demonstrated no structural or biochemical differences compared to the wild type enzyme, but contained a PEG molecule bound to the active site cavity, which allowed mapping of the residues forming this site. Our results demonstrated that PMGL2 and other known members of the GTSAG motif subfamily possess a common dimerization interface that can be a unique feature of this subfamily in contrast to the members of the GDSAG subfamily. Moreover, PMGL2 had a unique organization of the active site cavity with significantly different topology compared to the other lipolytic enzymes from family IV with known structure.

## Materials and methods

### Protein purification, crystallization and data collection

The gene coding for mPMGL2 was constructed by standard techniques and cloned into pET32a vector similar to the gene of wtPMGL2. Recombinant wtPMGL2 and its mutant form were expressed and purified as described in [21]. Biochemical characterization of the mutant was performed as described previously for the corresponding wild type protein [21].

The initial crystallization screening of wtPMGL2 and mPMGL2 was performed with a robotic crystallization system (Rigaku, USA) and commercially available 96-well crystallization screens (Hampton Research, USA) at 20°C using the sitting drop vapor diffusion method. The protein concentration was 14 mg/ml in 20 mM Tris, pH 8.0. The optimization of the initial crystallization conditions was done using the hanging drop vapor diffusion method. The optimized crystallization solution contained 250 mM MgCl_2_, 12% PEG3350 and 100 mM HEPES, pH 7.5. The same conditions were used for crystallization of the mutant form of the enzyme.

Immediately before data collection, the crystals of both PMGL2 forms were briefly soaked in the mother liquor containing 25% glycerol as a cryoprotectant. The crystals were then flash-cooled to 100 K in liquid nitrogen. The X-ray diffraction data were collected at the BL41XU beamline of a SPring8 synchrotron (Harima Science Garden, Japan). The data were indexed, integrated and scaled using iMOSFLM [34]. Based on the L-test [35], both datasets were not twinned. The program Pointless [36] suggested the P2_1_ space group.

The data collection and processing statistics are summarized in Table 1.

**Table 1:** Data collection, processing and refinement.

	wtPMGL2	mPMGL2
Diffraction source	BL41XU, Spring8	
Wavelength (Å)	1.0	1.0
Temperature (K)	100	
Detector	PILATUS	
Crystal-to-detector distance (mm)	210.00	250.0
Rotation range per image (°)	0.5	0.5
Total rotation range (°)	360	360
Space group	P2_1_	P2_1_
*a*, *b*, *c* (Å)	47.01, 92.35, 74.23	47.14, 92.18, 74.46
α, β, γ (°)	90.0, 106.83, 90.0	90.00 106.41 90.00
Average mosaicity (°)	0.74	0.71
Resolution range (Å)	56.31–1.60 (1.63–1.60)	56.46–1.43 (1.45–1.43)
Completeness (%)	97.4 (95.9)	98.0 (95.2)
Average redundancy	6.1 (5.9)	5.9 (5.5)
〈*I*/σ(*I*)〉	7.8 (2.9)	12.4 (1.8)
Rmeas (%) (Diederichs and Karplus 1997)	15.3 (71.6)	8.2 (1.0)
CC_1/2_ (Diederichs and Karplus 1997)	98.5 (83.3)	99.9 (62.6)
*R*_*fact*_ *(%)*	15.7	15.6
*R*_free._ *(%)*	18.7	18.4
Bonds (Å)	0.02	0.02
Angles (°)	2.30	1.92
Ramachandran plot		
Most favoured (%)	96.14	96.51
Allowed (%)	3.54	3.17
PDB entry code	6QIN	6QLA

### Structure solution and refinement

The structure of wtPMGL2 was solved at 1.60 Å resolution by the molecular replacement method using the MOLREP program [37] with the atomic coordinates of the esterase EstE5 from uncultured bacterium (PDB ID: 3L1H) as a starting model. The wtPMGL2 structure was then used to solve mPMGL2 at 1.43Å resolution by the MOLREP [37] program. The refinement of both structures was carried out using the REFMAC5 program of the CCP4 suite [38]. TLS was introduced at the earlier stages of refinement. The visual inspection of electron density maps and the manual rebuilding of the model were carried out using the COOT interactive graphics program [39]. The resolution was successively increased to 1.60 Å and 1.43 Å, correspondingly, and the hydrogen atoms in fixed positions were included during the final refinement cycles. In the final models, an asymmetric unit contained two independent copies (subunits A and B) of the protein. In the wtPMGL2 model one subunit contained 312 and the other had 318 visible residues together with total 590 water molecules, one chloride and two magnesium ions from the crystallization solution. In case of mPMGL2 an asymmetric unit contained two subunits each with 319 residues as well as one chloride and three magnesium ions, two PEG molecules from the crystallization conditions and 578 water molecules. Some N-terminal as well as C-terminal residues, including poly-His tag, were invisible in electron density of both structures possibly due to their high flexibility. In wtPMGL2 221–226 residues of the A molecule were also invisible in electron density, in contrast to the B molecule and mPMGL2 structure, where this region has a poor electron density. The superposition of two protein molecules within the same asymmetric units and between wtPMGL2 and mPMGL2 gave an RMSD between Cα atoms of approximately 0.3 Å, indicating the absence of large discrepancies between the molecules. Notably, the conformation of loops 202–209 and 216–237 was identical in wtPMGL2 and mPMGL2 (see below).

### Structure analysis and validation

The visual inspection of the structures was carried out using the COOT program [39] and the PyMOL Molecular Graphics System, Version 1.9.0.0 (Schrödinger, USA). Amino acid sequence alignment was made with BLAST [40] and Clustal Omega [41]. The structure comparison and superposition were made using the PDBeFold program [42], while contacts were analyzed using the PDBePISA [43] and WHATIF software [44].

## Results

### Overall structure of PMGL2 subunit

**Fig 1:**
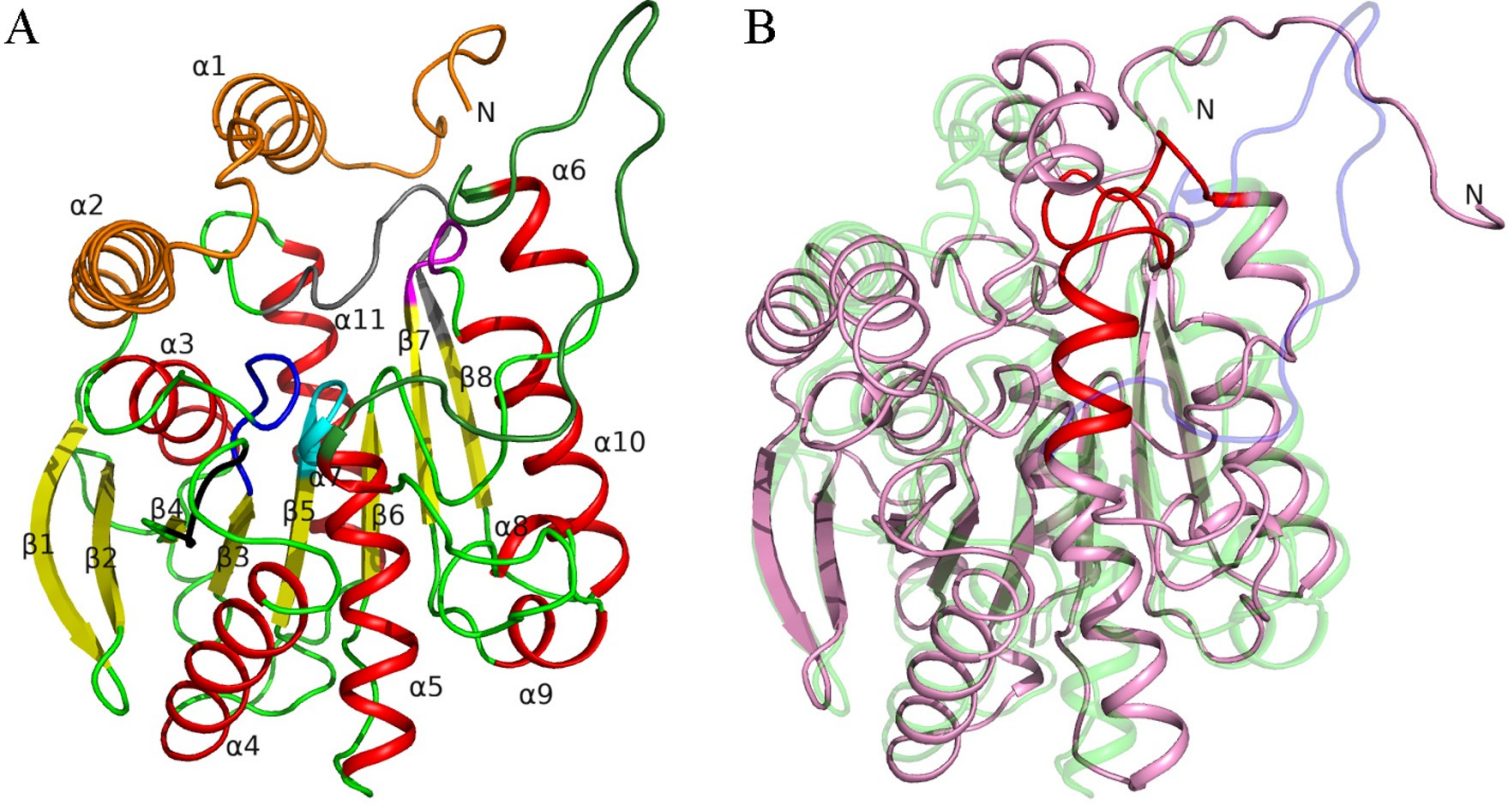
Structure of PMGL2 esterase. (A) Cartoon diagram of the wtPMGL2 subunit. Secondary structure elements are color coded (α-helix—red; β-strand—yellow; loop—green). Characteristic sequence motifs are highlighted as followed: ^105^HGGAF^109^(blue), ^269^GGRD^272^(magenta), ^296^FxxGxxHxxF^305^(grey), ^172^GCSAG^176^(cyan) and ^137^YRLA^140^(black). CAP-domain is highlighted in orange. The loop 216–237 is depicted in dark green. (B) Comparison of PMGL2 subunit (green, semitransparent) with homologous structure of esterase E25 from metagenomic library from the South China Sea (magenta, PDB entry 4Q05). Orientation of the wtPMGL2 is similar to panel A. Loop 216–237 of wtPMGL2 is highlighted in blue and corresponding loop of 4Q05 –in red.

Three-dimensional structures of subunits of wtPMGL2 and its mutant variant mPMGL2 were found to be quite similar (see Methods section). Both of them had a typical α/β bacterial HSL-like fold, consisting of two canonical domains ‒ a CAP-domain and a catalytic domain [16] (Fig 1A). The CAP domain (residues 1–68) contained two α-helices (α1 and α2). The large catalytic domain consisted of 8 β-strands (β1-β8) surrounded by 9 alpha-helices (α3-α11). Further in the text we will describe both structures together as PMGL2, if not stated otherwise.

**Fig 2:**
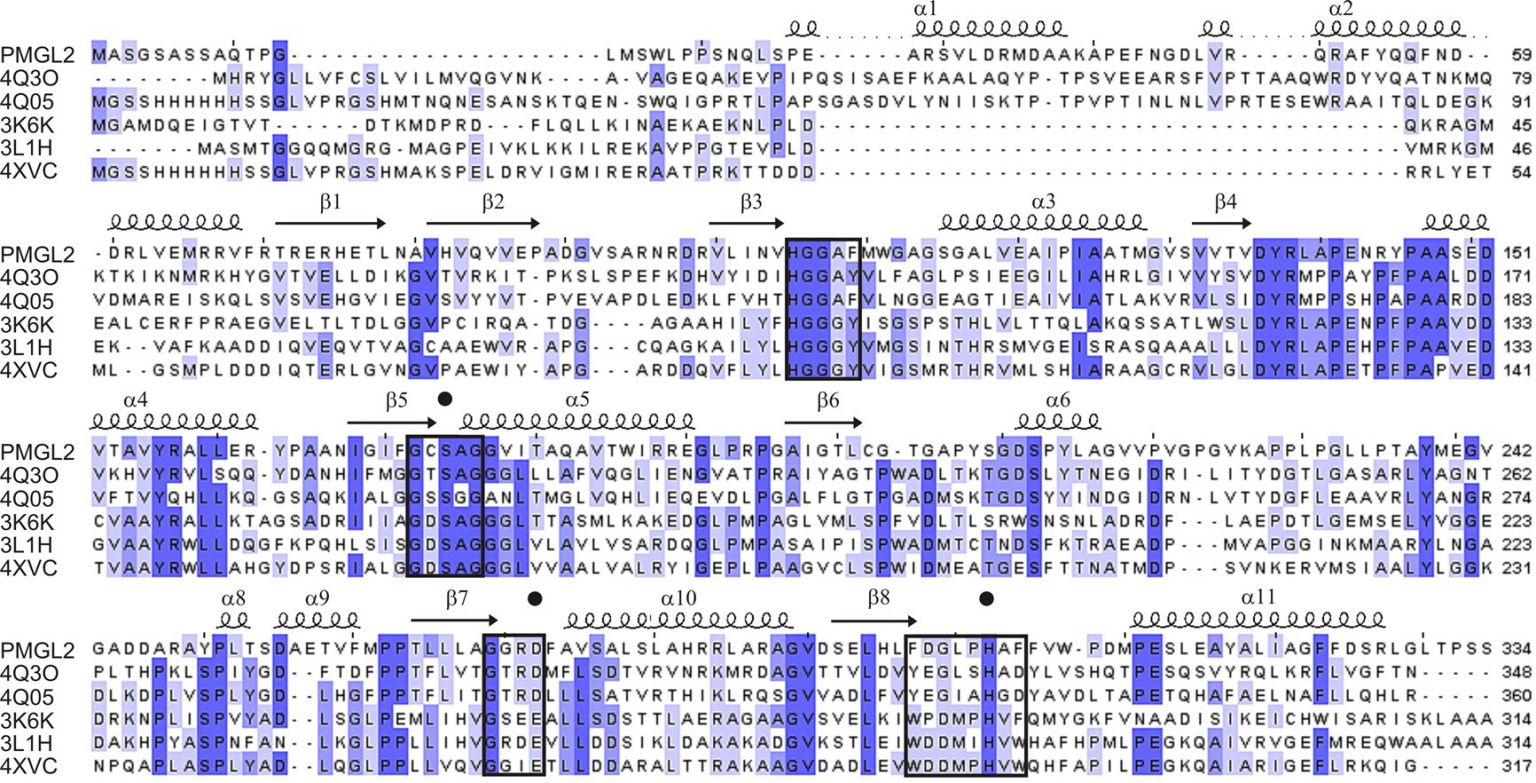
Multiple sequence alignment of PMGL2 and homologous enzymes with known three-dimensional structure. The extent of amino acid sequence conservation is depicted in grades of blue. PMGL2 secondary structure elements are indicated at the top. Black circles mark amino acid residues belonging to the catalytic triad.

Comparison of PMGL2 structure with those of homologue enzymes using PDBeFold revealed that despite the low sequence identity (Fig 2), the enzyme was structurally quite similar to bacterial HSL (bHSL) enzymes obtained from different metagenomic libraries (Table 2). Structure superposition demonstrated that secondary structure elements of PMGL2 subunit possessed nearly the same spatial positions as in the structures of homologous enzymes (Fig 1B). The major differences were found within the loops 202–209 and 216–237 of the catalytic domain. In case of PMGL2, these loops had strictly unique conformations in comparison with other known bHSLs (Fig 1B). In other bHSLs, the region corresponding to 216–237 in the PMGL2 is well ordered, harbors an extra α-helix and covers the active site cavity, forming numerous interactions with other parts of the protein subunit. In contrast, in PMGL2 this region was oriented off the protein, formed nearly no interactions with the rest of the protein subunit and thus dramatically widened the active site entrance (see below).

**Table 2:** Comparison of PMGL2 structure with the structures of homologous enzymes.

Enzyme	Sub-family	PDB code (reference through the text)	Q-score	RMSD, Å^2^	Superposed residues_, %_	Sequence identity, %
MGS-MT1 from a Lake Matapan deep-sea metagenomic library [45]	GTSAG	4Q3O	0.56	1.85	89	28
HSL-homolog EstE5 from a metagenomic library	GDSAG	3L1H	0.56	1.75	91	23
HSL-homolog EstE7 from a metagenomic library	GDSAG	3K6K	0.55	1.88	90	26
Esterase E40 from a marine sedimental metagenomic library [46]	GDSAG	4XVC	0.54	1.78	89	28
Esterase E25 from metagenomic library from the South China Sea [19]	GSSGG	4Q05	0.47	2.05	82	28

### Dimeric interface

**Fig 3:**
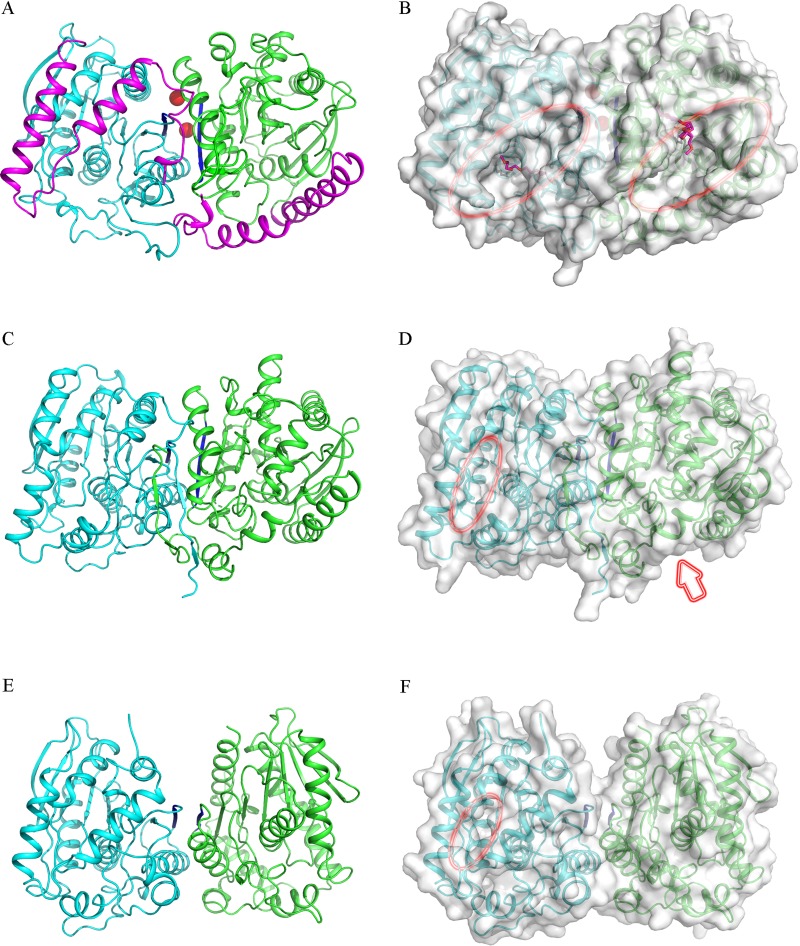
Comparison of the PMGL2 dimer with the dimers of homologous enzymes. (A) Cartoon representation of the mPMGL2 dimer. Enzyme subunits are highlighted in different colors. CAP-domains and β8 strands are colored in magenta and blue, respectively. Magnesium ions are shown as red spheres. (B) Surface representation of the mPMGL2 dimer. Hereinafter orientation of the molecules and color scheme are the same as on the panel A. PEG molecules bound in the active site cavity are depicted in pink. Entrances to the active site are marked with red ellipses, reflecting their approximate size. (C) Cartoon representation of the 4Q05 dimer. (D) Surface representation of the 4Q05 dimer. The second entrance to the active site on the side of the molecule is marked with an arrow. (E) Cartoon representation of the 3K6K dimer. (F) Surface representation of the 3K6K dimer. The second entrance to the active site is not marked as it is situated on the opposite side of the molecule. Note that in this case, β8 strands lie near in the same plane (are antiparallel).

Contact analysis of PMGL2 revealed that it exists as a homodimer in the crystal (Fig 3A), which was consistent with the results of gel-filtration studies (S1 Fig). The area that becomes buried upon PMGL2 dimer formation is 1421 Å^2^, which comprises about 11% of the total surface area of each subunit. According to PDBePISA, the dimeric interface was formed by 19 hydrogen bonds (residues 22–23, 209–210, 276, 282, 294 and 298), 17 salt bridges (residues 211, 271, 283, 287 and 315) as well as by hydrophobic interaction (residues 17–20, 214, 219–221, 230, 280, 286, 294–297 and 319), thus involving residues of CAP-domain, as well as α6, α10, α11 and β8 from each subunit (Figs 1A and 3A).

Interestingly, magnesium ions from the crystallization buffer additionally support the dimeric interface of PMGL2 in a crystal via hydrogen bonding of coordinated water molecules to adjacent protein subunits (Fig 3A). Treatment with 50 mM EDTA didn’t result in any significant changes of its thermal stability or chromatographic mobility (unpublished data). These data correlated with previous observation that the addition of magnesium ions or EDTA to 4Q05 had no effect on its enzymatic activity [19]. Moreover, Mg^2+^ was not revealed in the course of previous ultrahigh-resolution MALDI studies of the PMGL2 [21]. We have concluded that the presence of the magnesium ions in the protein structure was a consequence of their presence in the crystallization buffer and is not characteristic for the native protein.

Dimer formation is a characteristic feature of many esterases belonging to the bHSL family [19, 47, 48]. In major bHSLs with known dimeric (or oligomeric) structure, including HerE (PDB ID 1LZL) [47], Sto-Est (3AIK) [48], Est25 (4J7A) [49] and others, the dimeric interface is mainly formed by the interactions between two antiparallel β-strands–β8 from each of the subunits and doesn’t involve the CAP-domain [22, 50, 51]. 4Q05 was the first example of bHSL with a unique dimerization interface involving CAP-domain. In 4Q05 structure, β8 strands of adjacent subunits are also not antiparallel but rotated by ~280° relative to each other [19] (Fig 3C).

Besides PMGL2, 4Q05 is the only enzyme listed in Table 2 that has been proven to possess dimeric structure in solution [19]. However, structure analysis with PDBePISA demonstrated that two of the bHSLs from this list, namely 3K6K and 4Q3O, also had a dimeric structure at least in crystal. Consequent analysis of the corresponding dimeric structures revealed that PMGL2 and 4Q3O similarly to 4Q05 had dimerization interface with rotated (not antiparallel) β8 strands (Fig 3A and 3C). In addition, the dimerization interfaces of these enzymes included interacting residues from both the CAP- and the catalytic domains. On the other hand, the 3K6K dimer is organized similarly to the canonical dimers of bHSLs with antiparallel β8 strands and without involvement of the CAP-domain in dimerization interface (Fig 3E). Detailed analysis of the PMGL2 dimeric interface revealed a number of structural differences compared to 4Q3O and 4Q05. In PMGL2, the long loop 216–237 participated in dimerization by forming a number of hydrophobic interactions of residues Val219-Val221 with residues Trp17-Pro20 of the CAP-domain of the adjacent subunit (Fig 3A). The important consequence of the unique conformation of the loop 216–237 is an altered orientation of the active site entrances in PMGL2 dimer. Both active sites are oriented in one direction towards the dimer surface (Fig 3B), while in 4Q05 and 4Q3O their entrances are faced sideways (Fig 3D) and in 3K6K the entrances are oriented nearly to the opposite sides of the dimer (Fig 3F).

### Conserved bHSL motifs in PMGL2

The amino acid sequences of bacterial lipolytic enzymes belonging to the HSL family contain several conserved motifs [13, 18, 20, 52], which participate in the active site formation or are situated nearby. PMGL2 also shares these motifs, including ^105^HGGAF^109^, ^269^GGRD^272^, ^296^FxxGxxHxxF^305^ and others (Figs 1A and 2). Thus, motif HGGGF in the case of PMGL2 consists of ^105^HGGAF^109^. Motif GxSxG [13] or, more precisely, GTSAG motif, which is classical for this subfamily of bHSL [19] and contains the catalytic Ser174, is present in PMGL2 in a form of ^172^GCSAG^176^. Two other motifs [18], containing remaining residues of the catalytic triad, Asp272 and His302, are also present in PMGL2 with small modifications: ^269^GGRD^272^ and ^296^FxxGxxHxxF^305^. Additional YRLA-motif recently found in bHSLs [20] is conserved in PMGL2 (^137^YRLA^140^) and situated on the protein surface in the vicinity of the active site. A comparison with homologous enzymes demonstrated that this region in PMGL2 possesses the same conformation as in other homologue structures, including side-chain orientation. Notably, in case of 4Q3O and 4Q05 enzymes this motif looks like YRMP, where the proline residue is disturbing the conformation of the subsequent loop compared to PMGL2 and other two enzymes from Table 1.

### PMGL2 active site

In contrast to an overall spatial fold common within the bacterial HSL family, PMGL2 has important differences in its active site cavity organization. Thus, a protruding hydrophobic substrate channel which leads to PMGL2 active site is significantly more accessible from the protein surface in comparison with other bHSLs. This configuration results in the dramatically wider active site cavity in PMGL2 which is about 13х25 Å^2^ compared to 7х10 Å^2^ or less for homologous bHSLs (Fig 3B, 3D and 3F). The reason for this drastic difference is a unique conformation of the loops 202–209 and especially 216–237 of PMGL2 (Fig 1B).

**Fig 4:**
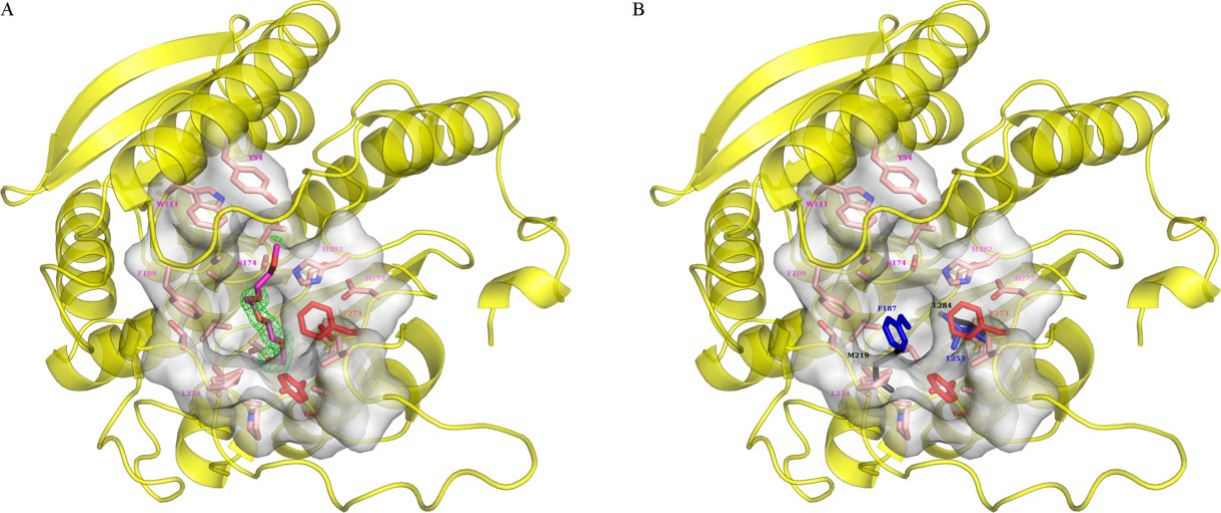
mPMGL2 active site entrance. (A) Amino acid residues of the PMGL2 that form the entrance are shown as semi-transparent grey surface and pink sticks and labeled in magenta. PEG molecule is shown in magenta together with its omit Fo-Fc map at 3σ level (green). Major residues restricting the active site cavity are shown and labeled in red. (B) Residues restricting active site cavity of 4Q05 (black) and 3K6K (blue) are superposed on PMGL2 structure from panel A.

The active site of PMGL2 consists of a catalytic triad of conserved His302, Asp272 and Ser174 (Figs 2, 4A and S2 Fig). The spatial arrangement as well as hydrogen bonds between Ser174-His302 and His302-Asp272 of this triad are very similar in all homologous structures, including PMGL2 [19, 53]. Motifs ^105^HGGAF^109^, ^172^GxSxG^176^, ^269^GGRD^272^ and ^296^FxxGxxHxxF^305^ comprise a part of the PMGL2 active site cavity and possess similar spatial position compared to homologous structures.

For unknown reasons, despite the same conformation of the loops 202–209 and 216–237 in both wtPMGL2 and mPMGL2 structures, a PEG molecule was found only in the active site of mPGML2 (Figs 3B and 4A). Each subunit of the asymmetric unit contains one PEG molecule bound in the active site cavity mainly via hydrophobic interactions. Key residues which participate in the cavity formation are: Thr204 and Ala206 which anchor PEG molecule by hydrogen bonds to its oxygen via their main chain, Gly107-Phe109, Thr173-Gly176, Val178, Ser202-Tyr208, Val218, Leu234-Thr236 and Asp272-Ala274. Residues Ala277-Leu278 comprise mainly hydrophobic surface surrounding the PEG molecule as well as aromatic Tyr54 and Trp111 that restrict the cavity from its top (Fig 4A). In the B subunit of mPMGL2, the PEG molecule was found in two conformations. The difference between these conformations was observed starting from the middle of the PEG molecule in a vicinity of the catalytic Ser174 and continued towards their C1 ends. The presence of two different PEG molecule conformations in the active site cavity most likely resulted from inefficient binding of this substrate analogue.

In all of the enzymes from Table 2, the active site cavity is sterically restricted on its bottom side by Phe or Leu, two large hydrophobic residues located in a position analogous to Ala274 of PMGL2 and by Trp or Phe residues from the loop corresponding to the loop 202–209 of PMGL2 (Fig 4B). It is noteworthy, that in the case of 4Q05 structure the latter restriction is achieved by Met219 residue [52] (Figs 2 and 4B). These restrictions lead to a narrower cavity in E25 esterase as compared to PMGL2. In the case of PMGL2, however, it has a unique active site size restriction that is accounted for by the Phe273 and Tyr208 side chains, with the latter resulting from the peculiar conformation of the 202–209 loop. In wtPMGL2, Tyr208 residue has two conformations of its side chain with relatively poor density. In contrast to wtPMGL2, in mPMGL2 the Tyr208 has one distinct conformation, resembling the one from the structure of wtPMGL2, which allows binding of the PEG molecule in the active site. This indicates that in the absence of substrate the side chain of Tyr208 is relatively flexible.

An additional residue, which might potentially affect the active site volume of bHSLs, is the one corresponding to Pro213 in PMGL2. It can decrease the active site cavity size being substituted for the residue with a large side chain, which takes place in e.g. 4Q3O (Leu234) and 4Q05 (Tyr226) (Fig 2).

### Effect of the point mutation in the GCSAG motif

**Fig 5:**
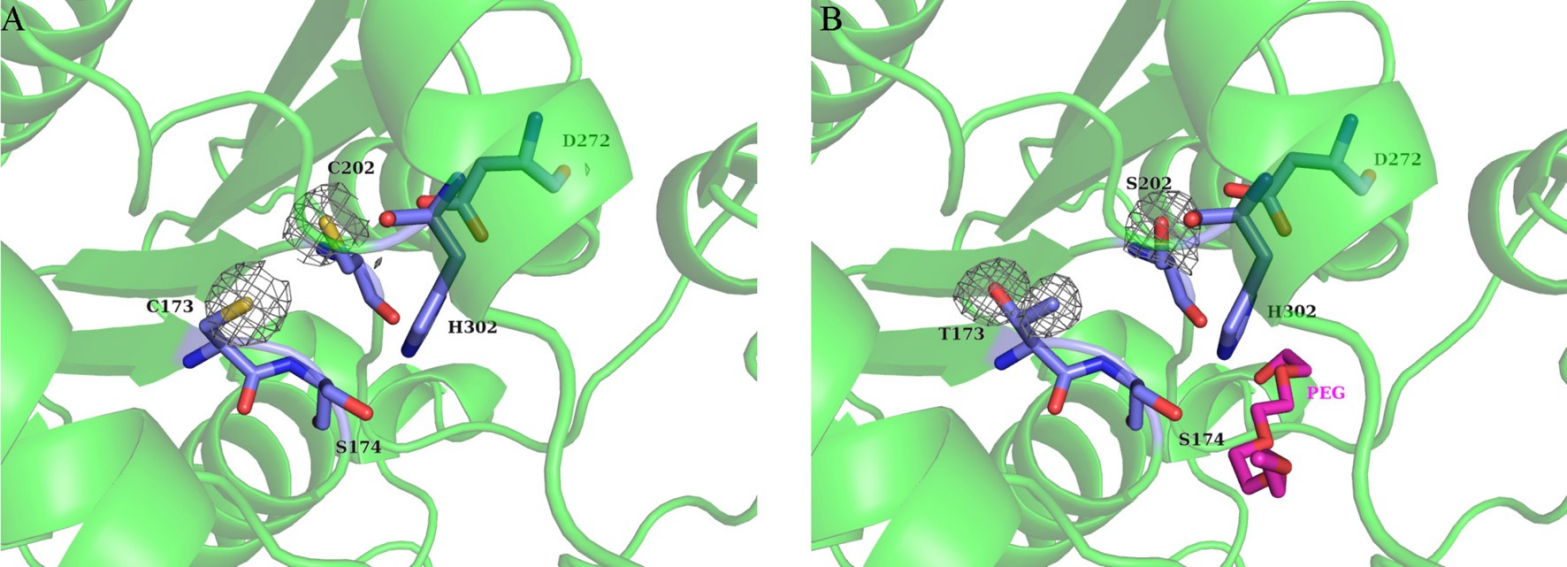
Residues of special interest in the PMGL2 active site. (A) Two cysteine residues in the wtPMGL2 active site with an appropriate omit Fo-Fc map at 3σ level (gray). Catalytic triad is also depicted. For clarity the rest of molecule is show as a semitransparent green cartoon. (B) The same region in the mPMGL2 active site containing mutations C173T and C202S with an appropriate omit Fo-Fc map at 3σ level. The bound PEG molecule is shown in magenta. The orientation and the color scheme are the same as on panel A.

Two cysteine residues, Cys173 and Cys202, were found in the active site of wtPMGL2, where Cys173 was a part of the ^172^GCSAG^176^ motif possessing the catalytic serine residue. The side chains of both cysteines were clearly visible in an electron density with a distance between their Cβ atoms of 5.2 Å (Fig 5A). However, these residues were not disulfide bonded in wtPMGL2 structure as followed from the electron density. The absence of the disulfide linkage in PMGL2 molecule was also confirmed in our previous work by ultrahigh-resolution MALDI analysis [21].

To unravel the effect of the unusual Cys173 on the properties of wtPMGL2, two point mutations were introduced in positions C173T and C202S resulting in mPMGL2 mutant variant. The second cysteine residue was mutated in order to avoid possible effects of its reactive group.

Gel-filtration analysis of the purified mPMGL2 demonstrated that it had the same elution profile as the wt protein (S1 Fig). The catalytic properties of mPMGL2 were examined at 25°C with *p*-nitrophenyl butyrate (C4) as a substrate. V_max_ and k_cat_ values for mPMGL2 were 22.3 μM/min and 4.6 s^-1^, respectively, and quite similar to V_max_ and k_cat_ values previously obtained for the wt protein (22.7 μM/min and 5.0 s^-1^) [21]. K_m_ value for mPMGL2 was 1.5 times higher than for wtPMGL2 (0.45 and 0.29 mM correspondingly).

In accordance with biochemical data, the structural analysis of mPMGL2 didn’t reveal any dramatic structural changes either, neither in the active site cavity nor in an overall fold of mPMGL2 in comparison with wtPMGL2. Both substituted residues were clearly seen in the electron density (Fig 5B). While Thr173 wasn’t forming any hydrogen bonds to the adjacent residues (except for the solvent molecule), the side chain of Ser202 participated in two new hydrogen bonds, one to a nitrogen atom of Ala303 and another one to OD2 atom of catalytic Asp272 (Fig 5B).

## Discussion

In this work, we have structurally characterized the PMGL2 esterase, a novel member of the GTSAG motif subfamily of the HSL family. The protein was obtained from the metagenomic DNA library constructed from the permafrost-derived microcosm. The PMGL2 demonstrated a fold typical for the HSL family of bacterial lipolytic enzymes with several unique structural features.

Similar to many esterases from the bHSL family, PMGL2 was a dimer in a crystal and in a solution. The dimerization interface was mainly formed by β8 strands of the adjacent subunits. Noteworthy, all three enzymes PMGL2, 4Q3O and 4Q05 with a non-classical dimerization interface (in which β8 strands are not antiparallel), fall into the non-GDSAG subfamily of bHSLs (Table 1). According to Li et al. [19], it could be speculated that such dimerization pattern is a common feature of the GTSAG subfamily of bHSLs. However, these results might just demonstrate a severe lack of the structural data on the members of the GTSAG subfamily of bHSLs. Besides the above mentioned enzymes, the only known structure of an enzyme from the GTSAG subfamily of bHSL is an esterase LipW from *Mycobacterium marinum* (PDB ID 3QH4), whose proposed dimer also shares the same structural feature [52]. It should be mentioned that this dimerization feature is sometimes found also in the lipases which do not belong to the bHSL family. For example, the dimerization interface of a tetrameric arylesterase from *Saccharolobus solfataricus* (PDB ID 5L2P) possessing the non-canonical GISAG motif is similar to a certain extent (non-antiparallel β8 strands) to that of the GTSAG subfamily.

The notable difference of PMGL2 subunit structure from homologous enzymes was in a strictly different conformation of the loops 202–209 and 216–237 belonging to the catalytic domain. Taking into account that the region 202–209 possesses the same orientation in wtPMGL2 and mPMGL2 and has relatively low thermal factor, it seems to have a functional conformation in a crystal. The region 216–237 also possesses the same conformation in both structures with, however, an absence of electron density for the residues 221–226 of one subunit of wtPMGL2 coupled with a high temperature factor and the poor electron density in this region in both structures. This could reflect relative flexibility of this loop (at least without proper substrate bound), and, thus, its unique conformation might be a result of a crystal packing. On the other hand, each of two PMGL2 subunits from the asymmetric unit has rather different crystal contacts in the vicinity of 216–237 region. In the structures of mostly homologous bHSLs, which are titled as "complex" (e.g. 3H19, 3H1B, 3G9Z, etc.), no bound substrate in the active site was found, except for PMSF molecule (e.g. 3H18), that is, however, not a real substrate. Nevertheless, in all homologous structures (either apo-enzyme or PMSF-complex) the loop corresponding to 216–237 in PMGL2 has closed conformation. Taking into account a bound PEG molecule in mPMGL2, is can be speculated that this loop is not mandatory for substrate binding. We might also suppose that this loop could possess a closed conformation in specific conditions within a cell. Noteworthy, insertions in a position structurally similar to loop 216–237 (as well as to the CAP-domain) of PMGL2 were found in the structures of a number of enzymes of various lipase families and proposed to have an important role in the proper substrate binding [53].

Different conformation of 202–209 and 216–237 loops in PMGL2 results in a significantly wider active site cavity in this enzyme. Interestingly, PEG molecule was found in the active site of mPMGL2 (but not in wtPMGL2). This finding makes mPMGL2 structure a unique example of family IV lipases with a bound ligand analogue, besides known structures with small inhibitors like PMSF. The ability of the enzyme to bind PEG molecule is attributed to a protruding cavity consisting of mostly hydrophobic residues. The size of the cavity correlates with the substrate specificity of the enzymes of this class in terms of size of the preferable substrates [54]. The length of PMGL2 active site cavity correlates with spatial parameters of middle-chain substrates (C8 and C10) which are specifically preferred by PMGL2 [21]. An interesting consequence of the unique 202–209 and 216–237 loops conformation is an alternative orientation of the PMGL2 active site compared to all structurally characterized enzymes of this family.

A unique feature of PGML2 is a threonine to a cysteine residue substitution in a characteristic motif GTSAG, resulting in the ^172^GCSAG^176^ motif. Similar substitutions were found in a number of the esterase sequences, but PMGL2 is the first example of a characterized enzyme from the HSL family possessing such motif. Previously, a thermostable lipase LipT with the same motif was obtained from the metagenomic library and biochemically characterized [53]. However, it belongs to the novel lipase family and demonstrates the temperature and substrate preferences which are completely different from PMGL2. To date, the functional role of this substitution is poorly understood. However, its possible role could become clearer taking into account the presence of another cysteine residue, Cys202, in a vicinity of Cys173 with a distance between Cβ atoms of these residues potentially allowing the disulfide bond formation. Biochemical and structural properties of mPMGL2 were very similar to the wild type protein. Moreover, structural studies and MALDI analysis did not reveal the presence of the disulfide bond between Cys202 and Cys173, however, it can be speculated that their proximity may allow for disulfide bonding in appropriate conditions and upon small movement of the main chain in the active site region. Interestingly, in the crystal structure of feruloyl esterase from *Aspergillus oryzae* (AoFaeB) belonging to the tannase family (PDB 3WMT, [55]) the catalytic Ser203 and His457 from the catalytic triad are connected by the disulfide bond between adjacent Cys202 and Cys458. Despite that the AoFaeB subunit displays moderate structural similarity to HSLs in general, and to PGML2 in particular (Q-score– 0.12, RMSD– 3.1 Å^2^), the position of the catalytic triad is similar. Structural analysis revealed that, in contrast to AoFaeB, the feasible disulfide bond Cys202-Cys173, if present in PMGL2, would fix the chain ^201^LSG^203^ within the active site without affecting the catalytic histidine. That indicates that in case of PMGL2 the feasible disulfide bond might have a different role, for example in stabilization of the active site of the enzyme by giving it some additional rigidity.

## Supporting information

S1 File Validation report for PDB code 6QIN. (PDF)

S2 File Validation report for PDB code 6QLA. (PDF)

S1 Fig Supplementary data. (DOCX)

S2 Fig Supplementary data. (DOCX)
