# Supplementary material for: Crystal structure of PMGL2 esterase from the hormone-sensitive lipase family with GCSAG motif around the catalytic serine
Source: PLoS One. 2020 Jan 28;15(1):e0226838. doi: 10.1371/journal.pone.0226838 (PMC6986724; doi:10.1371/journal.pone.0226838)
Supplement: S1 File — (PDF) [file pone.0226838.s001.pdf]

Full wwPDB X-ray Structure Validation Report (i)

CONFIDENTIAL VALIDATION REPORT

Jan 21, 2019 – 05:13 PM GMT

PDB ID	:	6QIN
Title	:	CRYSTAL STRUCTURE OF THE PMGL2 ESTERASE FROM PERMAFROST METAGENOMIC LIBRARY
Deposited on	:	2019-01-21
Resolution	:	1.60 Å(reported)

This is a Full wwPDB X-ray Structure Validation Report.

This report is produced by the wwPDB biocuration pipeline after annotation of the structure.

We welcome your comments at *validation@mail.wwpdb.org*
A user guide is available at
https://www.wwpdb.org/validation/2017/XrayValidationReportHelp
with specific help available everywhere you see the (i) symbol.

The following versions of software and data (see references (i)) were used in the production of this report:

MolProbity	:	4.02b-467
Xtriage (Phenix)	:	1.13
EDS	:	rb-20031633
Percentile statistics	:	20171227.v01 (using entries in the PDB archive December 27th 2017)
Refmac	:	5.8.0158
CCP4	:	7.0 (Gargrove)
Ideal geometry (proteins)	:	Engh & Huber (2001)
Ideal geometry (DNA, RNA)	:	Parkinson et al. (1996)
Validation Pipeline (wwPDB-VP)	:	rb-20031633

1 Overall quality at a glance (i)

The following experimental techniques were used to determine the structure:
X-RAY DIFFRACTION

The reported resolution of this entry is 1.60 Å.

Percentile scores (ranging between 0-100) for global validation metrics of the entry are shown in the following graphic. The table shows the number of entries on which the scores are based.

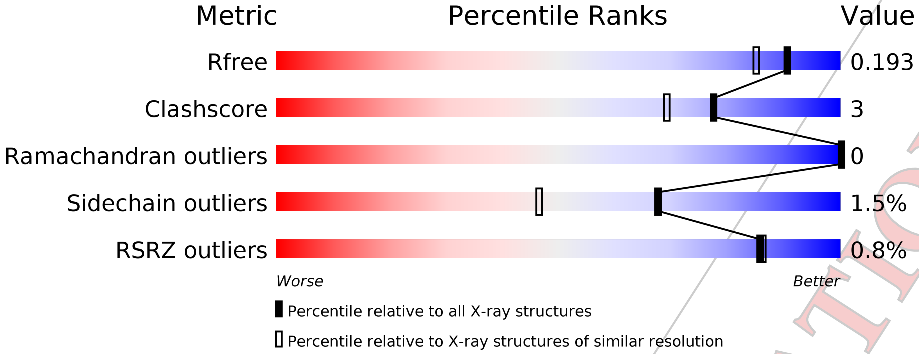

Metric	Whole archive (#Entries)	Similar resolution (#Entries, resolution range(Å))
R_{free}	111664	2957 (1.60-1.60)
Clashscore	122126	3202 (1.60-1.60)
Ramachandran outliers	120053	3117 (1.60-1.60)
Sidechain outliers	120020	3116 (1.60-1.60)
RSRZ outliers	108989	2883 (1.60-1.60)

The table below summarises the geometric issues observed across the polymeric chains and their fit to the electron density. The red, orange, yellow and green segments on the lower bar indicate the fraction of residues that contain outliers for >=3, 2, 1 and 0 types of geometric quality criteria. A grey segment represents the fraction of residues that are not modelled. The numeric value for each fraction is indicated below the corresponding segment, with a dot representing fractions <=5% The upper red bar (where present) indicates the fraction of residues that have poor fit to the electron density. The numeric value is given above the bar.

Mol	Chain	Length	Quality of chain
1	A	349	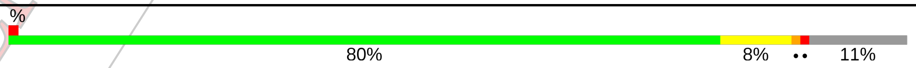
1	B	349	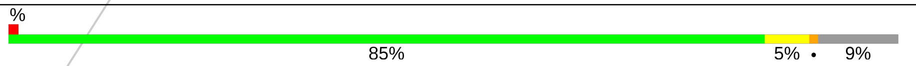

CONFIDENTIAL VALIDATION REPORT

2 Entry composition (i)

There are 4 unique types of molecules in this entry. The entry contains 5388 atoms, of which 0 are hydrogens and 0 are deuteriums.

In the tables below, the ZeroOcc column contains the number of atoms modelled with zero occupancy, the AltConf column contains the number of residues with at least one atom in alternate conformation and the Trace column contains the number of residues modelled with at most 2 atoms.

- Molecule 1 is a protein called PMGL2.

Mol	Chain	Residues	Atoms					ZeroOcc	AltConf	Trace
			Total	C	N	O	S			
1	A	312	2395	1512	436	437	10	0	10	0
1	B	318	2400	1520	426	444	10	0	4	0

There are 12 discrepancies between the modelled and reference sequences:

Chain	Residue	Modelled	Actual	Comment	Reference
A	344	HIS	-	expression tag	UNP A0A142J6I6
A	345	HIS	-	expression tag	UNP A0A142J6I6
A	346	HIS	-	expression tag	UNP A0A142J6I6
A	347	HIS	-	expression tag	UNP A0A142J6I6
A	348	HIS	-	expression tag	UNP A0A142J6I6
A	349	HIS	-	expression tag	UNP A0A142J6I6
B	344	HIS	-	expression tag	UNP A0A142J6I6
B	345	HIS	-	expression tag	UNP A0A142J6I6
B	346	HIS	-	expression tag	UNP A0A142J6I6
B	347	HIS	-	expression tag	UNP A0A142J6I6
B	348	HIS	-	expression tag	UNP A0A142J6I6
B	349	HIS	-	expression tag	UNP A0A142J6I6

- Molecule 2 is CHLORIDE ION (three-letter code: CL) (formula: Cl).

Mol	Chain	Residues	Atoms		ZeroOcc	AltConf
			Total	Cl		
2	A	1	1	1	0	0

- Molecule 3 is MAGNESIUM ION (three-letter code: MG) (formula: Mg).

Mol	Chain	Residues	Atoms		ZeroOcc	AltConf
			Total	Mg		
3	B	1	1	1	0	0

Continued on next page...

CONFIDENTIAL VALIDATION REPORT

Continued from previous page...

Mol	Chain	Residues	Atoms	ZeroOcc	AltConf
3	A	1	Total Mg<br>1 1	0	0

- Molecule 4 is water.

Mol	Chain	Residues	Atoms	ZeroOcc	AltConf
4	A	304	Total O<br>304 304	0	0
4	B	286	Total O<br>286 286	0	0

CONFIDENTIAL VALIDATION REPORT

3 Residue-property plots

These plots are drawn for all protein, RNA and DNA chains in the entry. The first graphic for a chain summarises the proportions of the various outlier classes displayed in the second graphic. The second graphic shows the sequence view annotated by issues in geometryand electron density. Residues are color-coded according to the number of geometric quality criteria for which they contain at least one outlier: green = 0, yellow = 1, orange = 2 and red = 3 or more. A red dot above a residue indicates a poor fit to the electron density (RSRZ > 2). Stretches of 2 or more consecutive residues without any outlier are shown as a green connector. Residues present in the sample, but not in the model, are shown in grey.

- Molecule 1: PMGL2

Chain A:

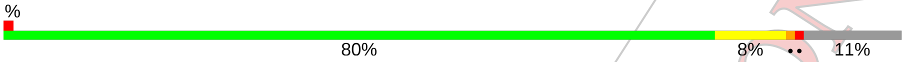

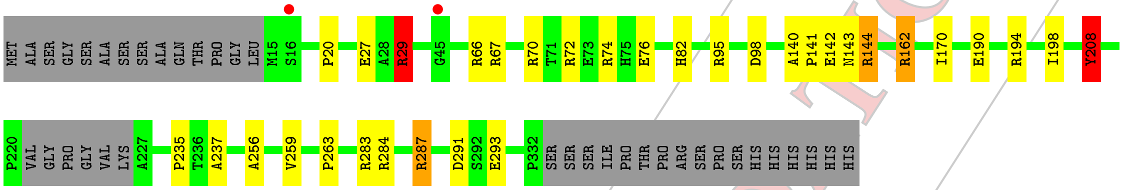

- Molecule 1: PMGL2

Chain B:

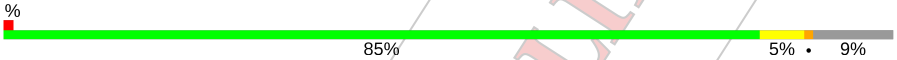

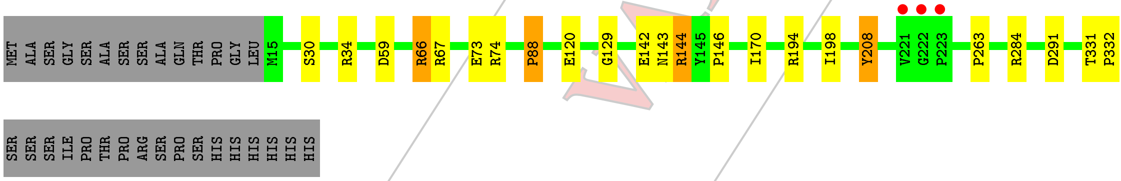

CONFIDENTIAL VALIDATION REPORT

4 Data and refinement statistics (i)

Property	Value	Source
Space group	P 1 21 1	Depositor
Cell constants<br>a, b, c, α, β, γ	47.01Å 92.35Å 74.23Å<br>90.00° 106.83° 90.00°	Depositor
Resolution (Å)	56.31 – 1.60<br>56.31 – 1.60	Depositor<br>EDS
% Data completeness<br>(in resolution range)	97.2 (56.31-1.60)<br>97.3 (56.31-1.60)	Depositor<br>EDS
R_{merge}	0.14	Depositor
R_{sym}	(Not available)	Depositor
$< I/\sigma(I) >$ [1]	2.86 (at 1.60Å)	Xtriage
Refinement program	REFMAC	Depositor
R, R_{free}	0.157 , 0.187<br>0.169 , 0.193	Depositor<br>DCC
R_{free} test set	3883 reflections (5.00%)	wwPDB-VP
Wilson B-factor (Å^2)	14.3	Xtriage
Anisotropy	0.153	Xtriage
Bulk solvent k_{sol}(e/Å^3), B_{sol}(Å^2)	0.34 , 46.4	EDS
L-test for twinning[2]	$< \|L\| >$ = 0.48, $< L^2 >$ = 0.31	Xtriage
Estimated twinning fraction	0.028 for h,-k,-h-l	Xtriage
F_o,F_c correlation	0.96	EDS
Total number of atoms	5388	wwPDB-VP
Average B, all atoms (Å^2)	19.0	wwPDB-VP

Xtriage's analysis on translational NCS is as follows: *The largest off-origin peak in the Patterson function is 6.10% of the height of the origin peak. No significant pseudotranslation is detected.*

[1] Intensities estimated from amplitudes.

[2] Theoretical values of $< |L| >$, $< L^2 >$ for acentric reflections are 0.5, 0.333 respectively for untwinned datasets, and 0.375, 0.2 for perfectly twinned datasets.

CONFIDENTIAL VALIDATION REPORT

5 Model quality (i)

5.1 Standard geometry (i)

Bond lengths and bond angles in the following residue types are not validated in this section: MG, CL

The Z score for a bond length (or angle) is the number of standard deviations the observed value is removed from the expected value. A bond length (or angle) with $|Z| > 5$ is considered an outlier worth inspection. RMSZ is the root-mean-square of all Z scores of the bond lengths (or angles).

Mol	Chain	Bond lengths		Bond angles	
		RMSZ	#$\|Z\|$ >5	RMSZ	#$\|Z\|$ >5
1	A	1.03	6/2508 (0.2%)	1.24	21/3419 (0.6%)
1	B	0.98	3/2481 (0.1%)	1.21	15/3388 (0.4%)
All	All	1.01	9/4989 (0.2%)	1.23	36/6807 (0.5%)

Chiral center outliers are detected by calculating the chiral volume of a chiral center and verifying if the center is modelled as a planar moiety or with the opposite hand.A planarity outlier is detected by checking planarity of atoms in a peptide group, atoms in a mainchain group or atoms of a sidechain that are expected to be planar.

Mol	Chain	#Chirality outliers	#Planarity outliers
1	A	0	2

All (9) bond length outliers are listed below:

Mol	Chain	Res	Type	Atoms	Z	Observed(Å)	Ideal(Å)
1	A	142	GLU	CD-OE1	7.01	1.33	1.25
1	A	208[A]	TYR	C-O	6.94	1.36	1.23
1	A	208[B]	TYR	C-O	6.94	1.36	1.23
1	B	120	GLU	CD-OE1	6.79	1.33	1.25
1	B	142	GLU	CD-OE2	-5.96	1.19	1.25
1	B	73	GLU	CD-OE2	-5.83	1.19	1.25
1	A	293	GLU	CD-OE2	5.74	1.31	1.25
1	A	67[A]	ARG	CB-CG	-5.04	1.39	1.52
1	A	67[B]	ARG	CB-CG	-5.04	1.39	1.52

All (36) bond angle outliers are listed below:

Mol	Chain	Res	Type	Atoms	Z	Observed(^{o})	Ideal(^{o})
1	B	208[A]	TYR	CB-CA-C	12.20	134.80	110.40
1	B	208[B]	TYR	CB-CA-C	12.20	134.80	110.40

Continued on next page...

CONFIDENTIAL VALIDATION REPORT

Continued from previous page...

Mol	Chain	Res	Type	Atoms	Z	Observed(°)	Ideal(°)
1	A	208[A]	TYR	CB-CA-C	10.32	131.05	110.40
1	A	208[B]	TYR	CB-CA-C	10.32	131.05	110.40
1	A	208[A]	TYR	CB-CG-CD1	9.50	126.70	121.00
1	A	208[B]	TYR	CB-CG-CD1	9.50	126.70	121.00
1	A	208[A]	TYR	CB-CG-CD2	-8.84	115.69	121.00
1	A	208[B]	TYR	CB-CG-CD2	-8.84	115.69	121.00
1	A	29[A]	ARG	NE-CZ-NH1	-8.12	116.24	120.30
1	A	29[B]	ARG	NE-CZ-NH1	-8.12	116.24	120.30
1	B	194	ARG	NE-CZ-NH2	-7.85	116.38	120.30
1	B	66	ARG	NE-CZ-NH2	-6.67	116.97	120.30
1	A	287[A]	ARG	CG-CD-NE	6.66	125.79	111.80
1	A	287[B]	ARG	CG-CD-NE	6.66	125.79	111.80
1	B	74	ARG	CB-CA-C	6.61	123.62	110.40
1	B	144[A]	ARG	CB-CG-CD	-6.36	95.08	111.60
1	B	144[B]	ARG	CB-CG-CD	-6.36	95.08	111.60
1	B	59	ASP	CB-CG-OD2	-6.13	112.78	118.30
1	A	284	ARG	NE-CZ-NH1	5.75	123.18	120.30
1	B	59	ASP	CB-CG-OD1	5.62	123.36	118.30
1	B	34	ARG	NE-CZ-NH1	5.50	123.05	120.30
1	B	30[A]	SER	CA-CB-OG	-5.44	96.51	111.20
1	B	30[B]	SER	CA-CB-OG	-5.44	96.51	111.20
1	A	144[A]	ARG	CG-CD-NE	-5.34	100.58	111.80
1	A	144[B]	ARG	CG-CD-NE	-5.34	100.58	111.80
1	B	284	ARG	NE-CZ-NH2	-5.31	117.64	120.30
1	A	194	ARG	CG-CD-NE	-5.28	100.72	111.80
1	A	95	ARG	NE-CZ-NH2	-5.28	117.66	120.30
1	A	98	ASP	CB-CG-OD1	-5.22	113.60	118.30
1	A	162	ARG	CB-CG-CD	5.14	124.96	111.60
1	A	283	ARG	NE-CZ-NH1	-5.12	117.74	120.30
1	A	66	ARG	NE-CZ-NH1	-5.04	117.78	120.30
1	A	66	ARG	CG-CD-NE	5.03	122.35	111.80
1	B	144[A]	ARG	CG-CD-NE	5.01	122.33	111.80
1	B	144[B]	ARG	CG-CD-NE	5.01	122.33	111.80
1	A	162	ARG	CG-CD-NE	5.00	122.30	111.80

There are no chirality outliers.

All (2) planarity outliers are listed below:

Mol	Chain	Res	Type	Group
1	A	235[A]	PRO	Mainchain
1	A	235[B]	PRO	Mainchain

CONFIDENTIAL VALIDATION REPORT

5.2 Too-close contacts (i)

In the following table, the Non-H and H(model) columns list the number of non-hydrogen atoms and hydrogen atoms in the chain respectively. The H(added) column lists the number of hydrogen atoms added and optimized by MolProbity. The Clashes column lists the number of clashes within the asymmetric unit, whereas Symm-Clashes lists symmetry related clashes.

Mol	Chain	Non-H	H(model)	H(added)	Clashes	Symm-Clashes
1	A	2395	0	2334	21	0
1	B	2400	0	2337	9	0
2	A	1	0	0	0	0
3	A	1	0	0	0	0
3	B	1	0	0	0	0
4	A	304	0	0	11	0
4	B	286	0	0	2	0
All	All	5388	0	4671	30	0

The all-atom clashscore is defined as the number of clashes found per 1000 atoms (including hydrogen atoms). The all-atom clashscore for this structure is 3.

All (30) close contacts within the same asymmetric unit are listed below, sorted by their clash magnitude.

Atom-1	Atom-2	Interatomic distance (Å)	Clash overlap (Å)
1:A:82[A]:HIS:CE1	4:A:505:HOH:O	1.93	1.17
1:A:190:GLU:HG2	4:A:638:HOH:O	1.73	0.88
1:A:72:ARG:HD3	4:A:515:HOH:O	1.82	0.80
1:A:74[B]:ARG:NH2	4:A:506:HOH:O	2.15	0.79
1:A:82[A]:HIS:NE2	4:A:505:HOH:O	2.10	0.73
1:A:20:PRO:HG2	1:A:29[A]:ARG:CZ	2.23	0.69
1:B:143:ASN:O	1:B:144[B]:ARG:HD3	1.92	0.68
1:A:143:ASN:O	1:A:144[A]:ARG:HD3	2.04	0.57
1:A:74[A]:ARG:NH1	1:A:76:GLU:OE2	2.37	0.57
1:A:20:PRO:HG2	1:A:29[A]:ARG:NH2	2.19	0.57
1:A:256:ALA:O	1:A:259:VAL:HG12	2.05	0.56
1:B:66:ARG:HH11	1:B:66:ARG:HG2	1.72	0.55
1:A:82[A]:HIS:CD2	4:A:505:HOH:O	2.57	0.55
1:A:27:GLU:HG2	4:A:742:HOH:O	2.08	0.54
1:A:208[A]:TYR:CD1	4:A:537:HOH:O	2.55	0.51
1:A:287[A]:ARG:NH1	4:A:504:HOH:O	2.39	0.50
1:A:208[A]:TYR:CE1	4:A:537:HOH:O	2.67	0.47
1:A:144[A]:ARG:HD2	1:A:237:ALA:O	2.16	0.46
1:A:263:PRO:HA	1:A:291:ASP:O	2.16	0.46
1:B:143:ASN:C	1:B:144[B]:ARG:HD3	2.35	0.46

Continued on next page...

CONFIDENTIAL VALIDATION REPORT

Continued from previous page...

Atom-1	Atom-2	Interatomic distance (Å)	Clash overlap (Å)
1:A:29[A]:ARG:HD3	1:A:29[A]:ARG:HH11	1.44	0.45
1:A:82[A]:HIS:CG	4:A:505:HOH:O	2.63	0.45
1:B:67:ARG:HD2	4:B:650:HOH:O	2.17	0.45
1:B:170:ILE:O	1:B:198:ILE:HA	2.17	0.44
1:B:67:ARG:CD	4:B:650:HOH:O	2.65	0.43
1:A:140:ALA:HB1	1:A:141:PRO:HA	2.01	0.43
1:B:263:PRO:HA	1:B:291:ASP:O	2.19	0.42
1:A:170:ILE:O	1:A:198:ILE:HA	2.21	0.41
1:B:88:PRO:HG3	1:B:129:GLY:HA2	2.01	0.41
1:B:331:THR:HA	1:B:332:PRO:HD3	1.91	0.41

There are no symmetry-related clashes.

5.3 Torsion angles (i)

5.3.1 Protein backbone (i)

In the following table, the Percentiles column shows the percent Ramachandran outliers of the chain as a percentile score with respect to all X-ray entries followed by that with respect to entries of similar resolution.

The Analysed column shows the number of residues for which the backbone conformation was analysed, and the total number of residues.

Mol	Chain	Analysed	Favoured	Allowed	Outliers	Percentiles	
1	A	318/349 (91%)	312 (98%)	6 (2%)	0	100	100
1	B	320/349 (92%)	317 (99%)	3 (1%)	0	100	100
All	All	638/698 (91%)	629 (99%)	9 (1%)	0	100	100

There are no Ramachandran outliers to report.

5.3.2 Protein sidechains (i)

In the following table, the Percentiles column shows the percent sidechain outliers of the chain as a percentile score with respect to all X-ray entries followed by that with respect to entries of similar resolution.

The Analysed column shows the number of residues for which the sidechain conformation was analysed, and the total number of residues.

CONFIDENTIAL VALIDATION REPORT

Mol	Chain	Analysed	Rotameric	Outliers	Percentiles	
1	A	247/271 (91%)	241 (98%)	6 (2%)	52	25
1	B	243/271 (90%)	239 (98%)	4 (2%)	65	44
All	All	490/542 (90%)	480 (98%)	10 (2%)	67	33

All (10) residues with a non-rotameric sidechain are listed below:

Mol	Chain	Res	Type
1	A	29[A]	ARG
1	A	29[B]	ARG
1	A	70	ARG
1	A	162	ARG
1	A	208[A]	TYR
1	A	208[B]	TYR
1	B	88	PRO
1	B	146	PRO
1	B	208[A]	TYR
1	B	208[B]	TYR

Some sidechains can be flipped to improve hydrogen bonding and reduce clashes. There are no such sidechains identified.

5.3.3 RNA (i)

There are no RNA molecules in this entry.

5.4 Non-standard residues in protein, DNA, RNA chains (i)

There are no non-standard protein/DNA/RNA residues in this entry.

5.5 Carbohydrates (i)

There are no carbohydrates in this entry.

5.6 Ligand geometry (i)

Of 3 ligands modelled in this entry, 3 are monoatomic - leaving 0 for Mogul analysis.

There are no bond length outliers.

There are no bond angle outliers.

CONFIDENTIAL VALIDATION REPORT

There are no chirality outliers.

There are no torsion outliers.

There are no ring outliers.

No monomer is involved in short contacts.

5.7 Other polymers ⓘ

There are no such residues in this entry.

5.8 Polymer linkage issues ⓘ

There are no chain breaks in this entry.

CONFIDENTIAL VALIDATION REPORT

6 Fit of model and data (i)

6.1 Protein, DNA and RNA chains (i)

In the following table, the column labelled '#RSRZ> 2' contains the number (and percentage) of RSRZ outliers, followed by percent RSRZ outliers for the chain as percentile scores relative to all X-ray entries and entries of similar resolution. The OWAB column contains the minimum, median, 95th percentile and maximum values of the occupancy-weighted average B-factor per residue. The column labelled 'Q< 0.9' lists the number of (and percentage) of residues with an average occupancy less than 0.9.

Mol	Chain	Analysed	<RSRZ>	#RSRZ>2			OWAB(Å^2)	Q<0.9
1	A	312/349 (89%)	-0.47	2 (0%)	89	89	9, 15, 35, 62	0
1	B	318/349 (91%)	-0.41	3 (0%)	84	84	9, 17, 39, 78	0
All	All	630/698 (90%)	-0.44	5 (0%)	86	86	9, 16, 36, 78	0

All (5) RSRZ outliers are listed below:

Mol	Chain	Res	Type	RSRZ
1	A	45	GLY	3.6
1	B	221	VAL	3.4
1	B	223	PRO	2.9
1	A	16	SER	2.7
1	B	222	GLY	2.6

6.2 Non-standard residues in protein, DNA, RNA chains (i)

There are no non-standard protein/DNA/RNA residues in this entry.

6.3 Carbohydrates (i)

There are no carbohydrates in this entry.

6.4 Ligands (i)

In the following table, the Atoms column lists the number of modelled atoms in the group and the number defined in the chemical component dictionary. The B-factors column lists the minimum, median, 95th percentile and maximum values of B factors of atoms in the group. The column labelled 'Q< 0.9' lists the number of atoms with occupancy less than 0.9.

CONFIDENTIAL VALIDATION REPORT

Mol	Type	Chain	Res	Atoms	RSCC	RSR	B-factors(Å^2)	Q<0.9
3	MG	B	401	1/1	0.98	0.08	17,17,17,17	1
3	MG	A	402	1/1	0.99	0.14	22,22,22,22	0
2	CL	A	401	1/1	1.00	0.07	11,11,11,11	0

6.5 Other polymers (i)

There are no such residues in this entry.

CONFIDENTIAL VALIDATION REPORT
