# Supplementary material for: Crystal structure of PMGL2 esterase from the hormone-sensitive lipase family with GCSAG motif around the catalytic serine
Source: PLoS One. 2020 Jan 28;15(1):e0226838. doi: 10.1371/journal.pone.0226838 (PMC6986724; doi:10.1371/journal.pone.0226838)
Supplement: S1 Fig — (DOCX) [file pone.0226838.s003.docx]

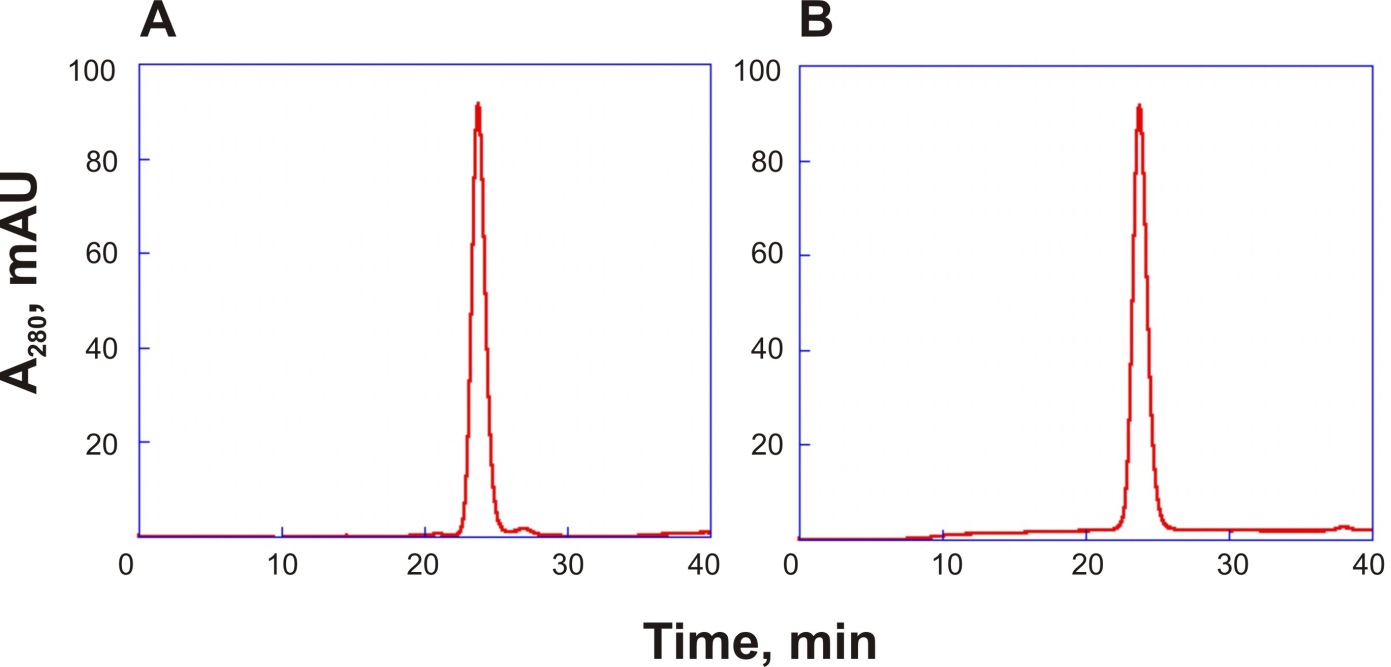


**S1 Fig. Supplementary data**. **Size exclusion chromatography of wtPMGL2 (A) and mPMGL2 (B).** Analytical gel-filtration was performed on a Superdex 75-10/300GL (GE Healthcare) column with flow rate 0.4 mL min^-1^ in 100 mM Tris-HCl, pH 8.0, 150 mM NaCl.
