# Supplementary material for: Crystal structure of PMGL2 esterase from the hormone-sensitive lipase family with GCSAG motif around the catalytic serine
Source: PLoS One. 2020 Jan 28;15(1):e0226838. doi: 10.1371/journal.pone.0226838 (PMC6986724; doi:10.1371/journal.pone.0226838)
Supplement: S2 Fig — (DOCX) [file pone.0226838.s004.docx]

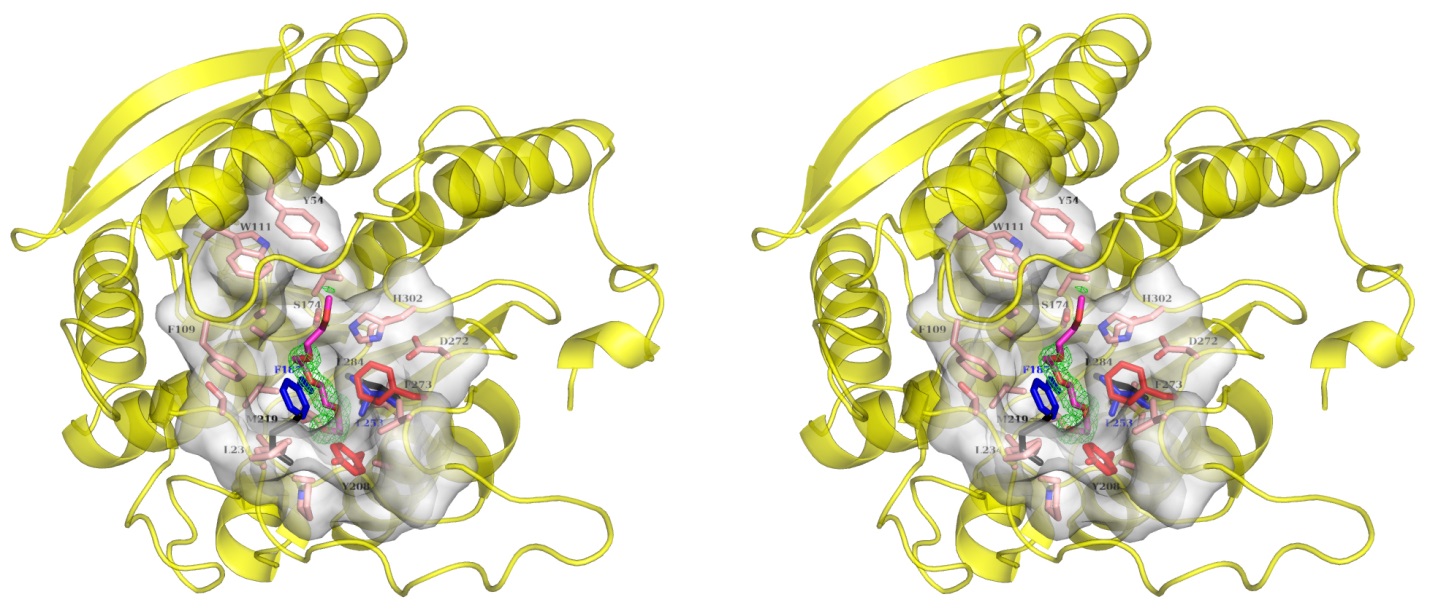


**S2 Fig. Supplementary data**. **Stereo-view of the mPMGL2 active site entrance.** Amino acid residues of the PMGL2 that form the entrance are shown as semi-transparent grey surface and pink sticks. PEG molecule is shown in magenta together with its omit Fo-Fc map at 3σ level (green). Residues restricting the active site cavity are shown in red (for PMGL2), black (4Q05) and blue (3K6K).
